# Membrane Potential: Accuracy and Reproducibility of Molecular Dynamics Simulations

**DOI:** 10.3390/membranes16070226

**Published:** 2026-07-01

**Authors:** Anna I. Malykhina, Svetlana S. Efimova, Olga S. Ostroumova

**Affiliations:** Institute of Cytology of Russian Academy of Science, Tikhoretsky Ave. 4, St. Petersburg 194064, Russia

**Keywords:** membrane dipole potential, molecular dynamics, force field parameterization, flavonoids, machine learning, finite-size effects, CHARMM, AMBER, Espaloma

## Abstract

The membrane dipole potential (*Ψ*_d_) is a critical modulator of ion transport and protein function, making the ability to accurately predict its modifications essential for rational drug design and membrane biophysics. While molecular dynamics (MD) simulations offer a powerful alternative to challenging in vitro experiments, their predictive accuracy is often hampered by sensitivities to simulation setups and force field parameterization. In this study, we provide a systematic evaluation of how system composition, box size, water models, and small-molecule parameterization protocols influence the calculated membrane potential. Using the CHARMM36m and AMBER (Lipid21) force fields, we demonstrate that CHARMM is notably more sensitive to box composition and finite-size effects than AMBER. We further show that the ~100 mV shift induced by 4-site water models is purely systematic; therefore, computationally efficient 3-site models remain reliable for predicting relative potential changes. Finally, we compare multiple parameterization strategies for three *Ψ*_d_-modifying flavonoids (baicalein, chrysin, luteolin) and show that standard CGenFF protocols fail to capture experimental trends, whereas ffTK-refinement and AMBER-based protocols (GAFF2 and Espaloma) significantly improve accuracy. Notably, the neural network-based Espaloma demonstrated surprisingly high predictive power, marking this approach as a promising, automated alternative for future studies. Our findings provide a set of practical recommendations for establishing reliable MD protocols to predict dipole potential modifications.

## 1. Introduction

The membrane dipole potential (*Ψ*_d_) is a fundamental biophysical parameter arising from the highly ordered orientation of molecular dipoles at the membrane-water interface. These dipoles primarily include lipid carbonyl groups at the glycerol backbone, zwitterionic or polar headgroup moieties, and structured interfacial water molecules [1]. Characterized by an immense electric field strength of approximately 10^8^–10^9^ V/m, *Ψ*_d_ significantly influences physiological processes by modulating ion permeability and the function of membrane-embedded proteins [2]. Specifically, the resulting positive potential barrier within the membrane interior explains why biological membranes are inherently more permeable to hydrophobic anions than to cations [3]. Beyond ion diffusion, the dipole potential acts as a key regulator of membrane protein conformational dynamics. Recent studies have shown that *Ψ*_d_ serves as a critical energetic barrier for the activation and deactivation kinetics of voltage-gated ion channels, such as the hERG channel [4]. Furthermore, the dipole potential profoundly influences the conductance of pore-forming peptides and antibiotics. For instance, plant-derived flavonoids and alkaloids modulate the lifetime and conductance of gramicidin A and syringomycin E channels specifically by reducing *Ψ*_d_ [5,6]. Consequently, the ability to predict modifications in the dipole potential is essential for effective drug design and development.

Molecular dynamics (MD) simulations have demonstrated high fidelity in capturing the structural organization of bilayers, showing excellent agreement with experimental data obtained from X-ray and neutron scattering [7]. However, predicting electrostatic parameters remains a significant challenge. This difficulty stems from two primary factors. First, the lack of a “gold standard” for comparison; measuring the dipole potential experimentally is notoriously difficult, resulting in widely varying values for identical bilayers [2,8]. Second, conventional force fields utilize fixed-charge models that ignore electronic polarizability. Although explicitly polarizable models (such as CHARMM Drude and AMOEBA) exist, they are computationally expensive and do not yet surpass the best nonpolarizable models in capturing membrane dynamics [9]. In practice, effective drug development does not require knowledge of the absolute *Ψ*_d_; instead, the focus is on the shifts caused by altering lipid composition or incorporating polar molecules.

The reliability of MD simulations of lipid bilayers is intrinsically linked to the choice of system size and box composition. It has been well-established that finite-size effects can significantly influence the physical properties of the membrane, such as lateral diffusion, bending moduli, and the long-range correlation of lipid motions [10,11]. These effects often arise from the suppression of long-wavelength undulations in smaller simulation boxes, which can lead to artifacts in the calculated electrostatic profile. Furthermore, the discrete nature of ions and water molecules in the solvent phase necessitates a precise stoichiometry to ensure reproducibility. Despite the extensive use of various force fields, there is a notable lack of systematic studies specifically comparing how minor fluctuations in box components—such as the addition of a single ion or slight changes in hydration—affect the membrane potential across different force fields. Understanding these sensitivities is crucial for establishing whether results obtained from moderate-sized systems can be reliably compared across different computational setups and force field parameters.

The accuracy of the membrane potential (*Ψ*) in MD simulations is highly dependent on the solvent model’s ability to represent the dielectric environment and interfacial water orientation. While the 3-site TIP3P model has been the historical standard due to its computational efficiency, it often fails to reproduce the correct electrostatic properties of the lipid–water interface [12]. Recent advancements have led to the adoption of more sophisticated 4-site models, such as TIP4P-Ew and OPC, which incorporate an additional virtual site to better distribute electronic charges. TIP4P-Ew was specifically parameterized to provide superior bulk properties when combined with Ewald summation techniques commonly used in the Charmm force field [12]. Similarly, the OPC model has been shown to offer optimal representations of water’s multipole moments, significantly improving the hydration of polar groups in Amber-based simulations [13]. Despite their growing popularity, the computational cost of 4-site models often limits their use in high-throughput studies of membrane-active molecules.

The accurate representation of small molecules in molecular dynamics simulations remains a significant challenge, as their force field parameters must precisely reproduce experimental physicochemical properties [14,15]. Currently, several automated parameterization frameworks are widely employed to streamline this process, most notably CGenFF (Comprehensive General Force Field) for the Charmm force field and GAFF2 (General Amber Force Field) for Amber. The fundamental difference between these two frameworks lies in their approach to partial charge assignment. CGenFF assigns charges by analogy, drawing from an extensive library of pre-optimized chemical fragments [16]. To refine such rule-based parameters, more rigorous protocols can be applied; for instance, the Force Field Toolkit (ffTK) is frequently used to optimize CGenFF parameters by fitting them to high-level quantum mechanical data, including molecular geometry, vibrational modes, and water interaction energies [17]. Alternatively, GAFF2 utilizes a more universal set of atom types and requires molecule-specific charge derivation—typically via AM1-BCC (Austin Model 1 with Bond Charge Corrections) or RESP (Restrained Electrostatic Potential) fitting—to capture the unique electrostatic environment of each ligand [18].

Beyond rule-based assignments, the field is rapidly evolving toward machine learning (ML) approaches, which represent a fundamental shift in how force field parameters are derived. By capturing complex, non-local electronic effects with ab initio accuracy, ML-driven potentials bypass the inherent limitations of fixed-charge models while remaining computationally feasible for large-scale simulations [19]. Specifically, it has been demonstrated that such models provide a superior description of structural and thermodynamic properties in molecular liquids by more accurately accounting for intricate intermolecular interactions compared to traditional frameworks [20]. Among these emerging methods, Espaloma (Extensible Surrogate Potential of ab initio Learned Objects and Molecular Alchemists) stands out as a particularly robust framework. Unlike many other ML models that focus solely on small molecule energetics, Espaloma utilizes graph neural networks to predict a complete set of force field parameters that are natively compatible with the functional forms of classical force fields like Amber [21].

To evaluate how accurately different parameterization strategies reproduce in vitro changes in the *Ψ*_d_, we selected three flavonoids with minimal structural variations that exert distinct effects on membrane electrostatics: baicalein (possessing three hydroxyl groups in the A-ring) has been shown to increase the dipole potential of DOPC membrane, while chrysin (with two hydroxyl groups in the same ring) and luteolin (featuring two hydroxyl groups in the A-ring and two in the B-ring) decrease it to varying degrees, with the effect being more pronounced in the latter [22]. Our previous study indicated that ffTK refinement of CGenFF parameters improved the predicted results for two out of three flavonoids [23]. However, as those findings relied on single replicates, the observed improvements might have been incidental due to inherent dipole potential fluctuations and trajectory noise. Moreover, a new version of CGenFF has recently become available [24]. Thus, it is worth investigating whether this new implementation overcomes the shortcomings of the previous version. The present study addresses these limitations by employing multiple independent replicates and introducing additional parameterization protocols for both the Amber force field and CGenFF v.5.

## 2. Materials and Methods

The initial three-dimensional structures of baicalein, chrysin, and luteolin were obtained from the PubChem database [25]. Automated parameter generation was performed using CGenFF (versions 4.6 and 5.0) [26]. For quantum-based parameterization, the Force Field Toolkit (ffTK v.2.1) plugin [27] for VMD and Gaussian 16 [28] were employed; the details of the ffTK parameterization protocol are described elsewhere [23]. GAFF2 [18] and Espaloma (v.0.3.2) [21] parametrizations were utilized for the Amber force field. Molecular dynamics (MD) simulations were performed using GROMACS 2026 [29] with the CHARMM36m all-atom [30] and Lipid21 Amber force fields [31]. Model membranes were constructed via the CHARMM-GUI Membrane Builder [32], each comprising 120 1,2-dioleoyl-*sn*-glycero-3-phosphocholine (DOPC) molecules. Flavonoids (baicalein, chrysin, luteolin) were embedded into the membrane headgroup region at lipid-to-modifier ratios of 10:1 and 5:1 (corresponding to 12 and 24 molecules, respectively). Each system contained 7200 water molecules with 11 K^+^ and 11 Cl^−^ ions, resulting in a 0.1 M KCl concentration.

Following energy minimization, a standard six-step equilibration protocol was employed to gradually release position restraints on the lipid molecules. Production runs were carried out at a constant temperature of 298.15 K (25 °C), maintained via a V-rescale thermostat with a 1.0 ps coupling constant. Pressure was kept at 1 bar using semi-isotropic coupling with the C-rescale barostat and a 5.0 ps time constant [33,34]. Long-range electrostatic interactions were treated with the Particle Mesh Ewald (PME) method [35], utilizing a 1.2 nm short-range cutoff. Van der Waals interactions were calculated using a Lennard-Jones potential with a force-switch modifier between 1.0 and 1.2 nm. Visualization was performed using VMD [36]. The dipole moments of flavonoids were calculated and visualized using the VMD Dipole Moment Watcher plugin. The membrane dipole potential was determined using the gmx potential GROMACS.

To ensure the statistical significance of the observed differences in membrane potential, we employed a multi-stage analytical approach. For each condition, four independent replicates were performed. Each simulation was run for 200 ns, with the first 20 ns discarded as equilibration. The analysis was conducted on the subsequent 180 ns production phase, with potential values sampled every 10 ps, yielding 1800 data points per replicate.

To evaluate the impact of box composition on membrane potential, we employed Linear Mixed-Effects Models using the statsmodels library in Python 3.12.2. This approach was chosen to account for the hierarchical structure of the MD data and the intrinsic autocorrelation within time series. In the model, hydration level and ion concentration were treated as fixed effects, while the individual replicates were nested within conditions as a random effect. The model was fitted using the Restricted Maximum Likelihood (REML) method to provide unbiased estimates of variance components. This allowed us to partition the total variance into two scales: the residual scale, representing intra-trajectory thermal fluctuations, and the group variance, representing the stochasticity between independent simulation runs. To assess the effect of system size, the number of lipids was varied to 60, 120, 240, and 480 lipids per system. Each system was hydrated with 60 water molecules per lipid, and the ion concentration was maintained at 0.1 M KCl in all cases. A one-way ANOVA with Tukey’s post hoc test was performed on the ensemble-averaged values.

The immersion depth of the flavonoid molecules within the lipid bilayer was determined by calculating the center-of-mass distance between each individual modifier molecule and the bilayer center along the z-axis using the gmx distance tool from the GROMACS package. For each individual simulation trajectory, the immersion depth values were averaged over all analyzed frames and across all constituent molecules of the system. The final values presented in the study represent the grand mean calculated across four independent simulation replicates, accompanied by the corresponding standard deviation (mean ± SD) to characterize the statistical uncertainty and system reproducibility.

To characterize the spatial orientation of the flavonoids within the lipid bilayer, the tilt angle (θ) of their aromatic core relative to the membrane normal (z-axis) was calculated. The molecular axis was defined by a vector connecting two characteristic core atoms, with raw angles extracted using the gmx gangle tool from the GROMACS package. To ensure that 0° corresponds to a vertical orientation and 90° to a molecule lying flat on the membrane surface, a directional correction (θ_corrected = 180° − θ_raw) was applied to the upper monolayer to counteract the symmetric nature of the bilayer. Molecules undergoing inter-monolayer transition (flip-flop) events were completely excluded from the statistical analysis to prevent equilibrium data distortion. To avoid geometric artifacts from three-dimensional fluctuations, the average orientation for each trajectory was computed trigonometrically via the mean cosine as ⟨θ⟩ = arccos(⟨cos θ_corrected⟩). The final values reported represent the grand mean calculated across four independent simulation replicates, accompanied by the corresponding standard deviation (mean ± SD) to evaluate the system’s reproducibility and thermal mobility.

The number of intermolecular contacts between the flavonoid molecules was evaluated using the Hydrogen Bonds plugin in the VMD 2.0 software. The calculation included all inter-flavonoid contacts within a distance cutoff of 6 Å, thereby capturing both hydrogen bonding and van der Waals interactions. For each simulation, the contact number was averaged over the analyzed trajectory. The final values presented in the study represent the grand mean calculated across four independent simulation replicates, accompanied by the corresponding standard deviation (mean ± SD).

## 3. Results and Discussion

### 3.1. Sensitivity of Membrane Electrostatics to System Composition

There are various methods for assembling membrane systems. For some approaches, such as manual assembly, precisely controlling the number of water molecules or ions can be challenging. Furthermore, there is no strict standard for the volume of the aqueous phase; current protocols, such as the CHARMM-GUI Membrane Builder [37], typically recommend a minimum water layer thickness of 2.25 nm on each side of the bilayer to avoid periodic boundary artifacts. Nevertheless, for investigations specifically focusing on the *Ψ*, maintaining a significantly larger aqueous phase is highly recommended, with 50–60 waters per lipid being a widely accepted literature standard [38]. A thicker water layer ensures that bulk-like water properties and dielectric behavior are fully restored in the center of the box, which effectively prevents unphysical electrostatic interactions between the periodic images of the bilayer and allows for the proper, unconstrained solvation of ions. This raises a practical question of how sensitive the calculated potential is to minor fluctuations within this optimal hydration range.

**Figure 1:**
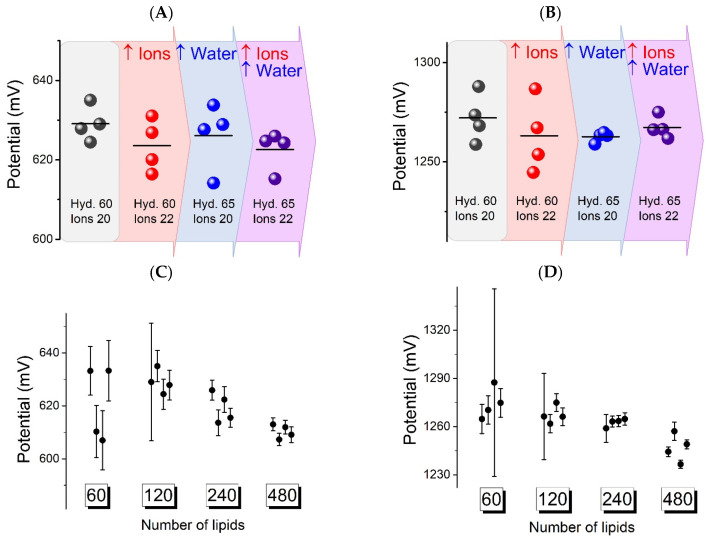
Comparison of DOPC membrane potential across different simulation conditions for Charmm (**A**,**C**) and Amber (**B**,**D**) force fields. (**A**,**B**) Figure shows 120-lipid systems with varying hydration (60 or 65 waters/lipid) and ion counts (10 or 11 ions of either sign). Solid black lines represent the ensemble average for each condition, while spheres indicate the mean values of four independent replicates. (**C**,**D**) Systems with sequentially doubled sizes ranging from 60 to 480 lipids (hydration of 60 waters/lipid, 0.1 M KCl). Replicates are shown as mean values; error bars represent statistical error estimates for individual trajectories.

To evaluate this sensitivity and the overall reliability of *Ψ* measurements, we performed a comparative statistical analysis of two widely used force fields, Charmm (CHARMM36M) and Amber (Lipid21). We investigated how minor variations in hydration (60 vs. 65 water molecules per lipid) and ion count (10 vs. 11 ions of either sign) influence the results across multiple independent replicates (Figure 1A,B). The mean potential values of the DOPC membrane system for all conditions are summarized in Table 1.

The impact of box composition was analyzed using Linear Mixed-Effects Models (LMM) to account for the nested structure of the data (Table 2). The LMM revealed that the residual scale (representing intra-trajectory fluctuations) accounted for the vast majority of the variance, while the group variance (representing differences between independent replicas) was negligible. This indicates high convergence of the MD trajectories and suggests that a 180 ns sampling window is sufficient to accurately determine the potential, making the results highly reproducible across independent runs. Although neither force field showed statistically significant shifts for hydration level, ion concentration, or their interaction (*p* > 0.05), Charmm exhibited a higher sensitivity to minor compositional changes compared to Amber. This is evidenced by the substantially lower *p*-values for ion concentration (*p* = 0.157 vs. 0.925) and a more pronounced effect size relative to the baseline potential (Figure 1).

The impact of the simulation box size was assessed by comparing systems with sequentially doubled sizes ranging from 60 to 480 lipids (hydration of 60 waters/lipid, 0.1 M KCl) (Figure 1C,D, Table 1). Although the ANOVA test revealed statistically significant differences for both force fields, the magnitude of these differences varied substantially (Charmm: *p* = 0.001, Amber: *p* = 0.046). Tukey’s post hoc pairwise comparisons showed that the 480-lipid system differed significantly from all other sizes in Charmm (*p* < 0.001 vs. 60 lipids, *p* = 0.007 vs. 120 lipids, and *p* = 0.038 vs. 240 lipids), whereas for Amber, a significant difference was observed only between the 480 and 120-lipid systems (*p* = 0.03). A downward trend in the *Ψ* upon system size increase was observed in both force fields, though it was more pronounced in Charmm. This suggests that Amber is overall less sensitive to box-size scaling artifacts regarding membrane potential than Charmm.

The physical origin of the statistically significant finite-size effect observed in the Charmm force field was further investigated by comparing the structural parameters of the systems. Analysis of the average area per lipid (APL) revealed that both force fields maintain remarkable structural stability upon scaling. The average APL upon membrane size increase were 67.04 ± 0.28 Å^2^, 66.91 ± 0.28 Å^2^, 66.96 ± 0.17 Å^2^, and 67.06 ± 0.24 Å^2^ for Charmm and 67.59 ± 0.30 Å^2^, 67.52 ± 0.23 Å^2^, 67.43 ± 0.16 Å^2^, and 67.5 ± 60.11 Å^2^ for Amber for the 60, 120, 240, and 480 lipid systems, respectively. This negligible difference in packing (less than 0.2%) confirms that the observed divergence in *Ψ* is not driven by altered lipid density or lateral pressure artifacts. Furthermore, the fact that both force fields yield nearly identical APL values (~67.0–67.5 Å^2^) highlights that the twofold difference in their absolute *Ψ* (0.63 V vs. 1.27 V) is a direct consequence of their electrostatic parameterization rather than differences in lipid organization. In Amber, the absolute *Ψ* baseline is inherently inflated due to its vacuum-based RESP charge derivation strategy, as established in earlier force field development studies [39,40]. This protocol fixes over-polarized partial charges on lipid atoms to implicitly compensate for the lack of explicit electronic polarization in a condensed phase. However, because this electrostatic overestimation is uniform and spatially tied to the identical structural organization of the bilayer, it operates as a purely systematic error. When evaluating relative changes, this artificial baseline contribution perfectly cancels out, which makes the Amber force field a fully reliable tool for determining changes in membrane dipole potential.

The observed decrease in the *Ψ* for the Charmm force field upon scaling the system, despite the lack of significant structural changes, can be attributed to PME-related finite-size effects. According to Hub et al. [41], Ewald-based methods introduce specific electrostatic artifacts in heterogeneous systems with sharp dielectric jumps, such as the water-lipid interface. These artifacts are sensitive to the simulation box geometry and the magnitude of the dipole density. The significantly higher partial charges in the Charmm force field—specifically on the phosphate phosphorus (+1.50 vs. +1.34 in Amber), phosphate oxygens (−0.78 vs. −0.51), and the choline nitrogen (−0.60 vs. +0.25)—create a much more intense interfacial dipole. Consequently, the Charmm system exhibits a stronger coupling with its periodic images in the smaller box, leading to an artificially elevated potential that relaxes as the box size increases. At the same time, the more moderate charge distribution in Amber appears to reach electrostatic convergence already at the 120-lipid scale. In summary, while finite-size effects are present in both force fields and are more pronounced in Charmm, the *Ψ* shift itself is relatively small. Thus, the *N* = 120 system provides a stable and reproducible measurement of the *Ψ* within its own configuration.

### 3.2. Sensitivity of Membrane Electrostatics to Water Model

**Figure 2:**
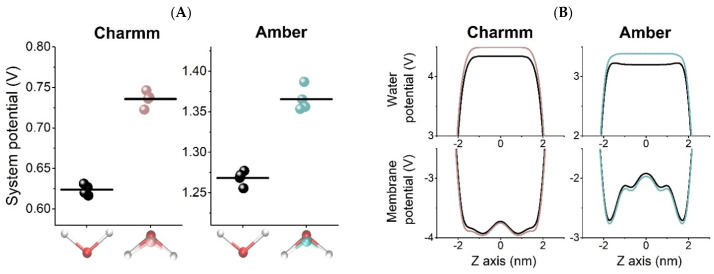
Comparison of membrane dipole potential across different water models and force fields. (**A**) Influence of water model on the average dipole potential in Charmm (TIP3P vs. TIP4P-Ew) and Amber (TIP3P vs. OPC) force fields. Large black horizontal lines represent the ensemble average for each condition, while spheres indicate the mean values of four independent replicates. Statistical significance was determined at *p* < 0.001. (**B**) Profiles of the electrostatic potential along the bilayer normal (Z-axis) decomposed into membrane and solvent contributions. The 3-site model (TIP3P) is shown as a black line, while 4-site models are shown in dark pink in Charmm and cyan in Amber.

The transition from 3-site to 4-site water models resulted in a consistent and statistically significant increase in the *Ψ* for both force fields (*p* < 0.001). In the Charmm system, replacing TIP3P with TIP4P-Ew shifted the mean *Ψ* from 624 ± 7 mV to 736 ± 10 mV (a net increase of ~112 mV). Similarly, in the Amber system, the use of the OPC model led to a shift from 1268 ± 9 mV to 1365 ± 15 mV (a net increase of ~97 mV) (Figure 2A). Decomposition of the total electrostatic potential into lipid and solvent contributions reveals that the shift observed upon transitioning to 4-site water models is almost entirely driven by the solvent phase (Figure 2B).

The most prominent difference between force fields in Figure 2B is the sharp drop of the electrostatic potential toward the hydrophobic core (center of the membrane, z = 0) observed in Amber, contrasted with the plateau-like behavior in Charmm. This feature is a well-documented artifact inherent to the different parameterization strategies of lipid aliphatic tails in classical force fields [42,43]. In Amber, the specific partial charge assignment and Lennard-Jones parameters of the terminal methyl and methylene groups lead to a strong local electric field toward the end of the acyl chains. Near the bilayer center, these fields from opposing leaflets significantly cancel each other out, resulting in a characteristic local potential drop. In contrast, Charmm balances headgroup-to-tail electrostatic contributions differently, maintaining a higher potential baseline within the hydrophobic interior. The difference in the lipid profile between the Amber and Charmm force fields is well-known and has been previously described [42]. While the membrane contribution remains nearly identical across water models in both force fields, the refined water models (TIP4P-Ew and OPC) exhibit a significantly higher positive *Ψ* for solvent, which accounts for the overall increase in the system’s potential at the bilayer center. The remarkable agreement in the magnitude of this shift (~100 mV) suggests that the underlying lipid–water electrostatic coupling responds systematically to the refined treatment of water’s dipole and quadrupole moments, regardless of the specific force field parameters. These results validate our use of the TIP3P model for studying dipole modifiers; since the shift induced by the water model is constant across different force fields, we expect the relative effects of modifiers to be additive and preserved, regardless of the absolute baseline potential.

### 3.3. Sensitivity of Membrane Electrostatics to Dipole Modifiers

**Figure 3:**
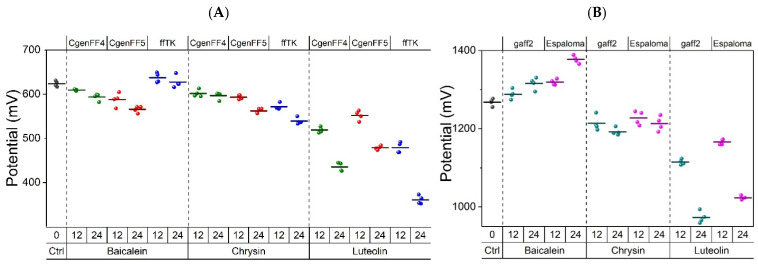
Comparison of predicted DOPC membrane dipole potential in the presence of flavonoids for (**A**) Charmm and (**B**) Amber force fields using different parameterization protocols. Horizontal black bars represent the ensemble average for each condition, while spheres indicate the mean values of the four independent replicates. For each force field, the first condition (leftmost) represents the control pure lipid bilayer. Subsequent data points correspond to baicalein, chrysin, and luteolin at two concentrations (12 and 24 molecules per system).

The evaluation of dipole modifiers introduces additional complexity, as there is currently no consensus on the optimal parameterization strategy for small molecules. To address this, we compared five distinct protocols: CGenFF (versions 4.6 and 5.0) and ffTK refinement for the Charmm force field, alongside GAFF2 and the machine-learning-based Espaloma for the Amber force field. We selected three flavonoids with minimal structural variations but divergent effects on the *Ψ*. For each compound, simulations were performed at two concentrations (12 and 24 molecules), with the modifiers initially embedded in the membrane at the level of the lipid glycerol groups. Each system was simulated in four independent replicates to ensure that the observed electrostatic changes were consistent and not biased by individual trajectory fluctuations. The obtained results are summarized in Table 3 and Figure 3.

To evaluate the predictive power of the tested force fields, we compared the calculated Δ*Ψ* against experimental data, using control values of 623.6 mV for Charmm and 1268.1 mV for Amber (the control values for 60 water molecules per lipid and 22 ions, as these parameters were used in all simulations with modifiers). For baicalein, which experimentally increases the potential by 36 ± 11 mV [22], only the Amber-based protocols successfully reproduced the direction of the shift. At a concentration of 12 molecules, GAFF2 yielded 1288 ± 12 mV (Δ*Ψ* ≈ +20 mV) and Espaloma showed 1319 ± 7 mV (Δ*Ψ* ≈ +51 mV). In contrast, all Charmm-based protocols, including both CGenFF versions and ffTK refinement, failed to capture this trend, resulting in values ranging from 588 to 637 mV, which effectively correspond to a neutral or slightly negative impact. In the case of chrysin, which exerts a moderate experimental decrease of −52 ± 5 mV [22], both Amber and refined Charmm protocols showed high accuracy. The ffTK refinement in Charmm demonstrated excellent agreement with experiment, yielding 572 ± 7 mV (Δ*Ψ* ≈ −52 mV) at 12 molecules, while GAFF2 produced a nearly identical shift to 1214 ± 19 mV (Δ*Ψ* ≈ −54 mV). Standard CGenFF v.4 and v.5 protocols were less sensitive, underestimating the effect with a decrease of only −20 to −30 mV. Luteolin exhibited the most pronounced experimental effect (−116 ± 10 mV [22]), and all tested protocols correctly identified it as the strongest modifier. At 12 molecules, Espaloma provided the closest match to experimental values with 1166 ± 7 mV (Δ*Ψ* ≈ −102 mV), while ffTK and GAFF2 predicted slightly larger shifts of −145 mV and −153 mV, respectively. Doubling the concentration to 24 molecules led to a drastic drop in potential across all models, with ffTK reaching 361 ± 10 mV (Δ*Ψ* ≈ −262 mV), indicating a high sensitivity of these parameterization strategies to luteolin concentration.

Our results suggest that a concentration of 12 molecules per membrane is sufficient to capture the characteristic trends of *Ψ* modification for the studied flavonoids. This concentration provides a clear differentiation between the compounds while maintaining a high signal-to-noise ratio. Increasing the concentration to 24 molecules did not qualitatively change the results for baicalein and chrysin, although it led to an exaggerated response in the case of luteolin, potentially overestimating its experimental impact. Overall, the results demonstrate that while Amber-based protocols (GAFF2 and Espaloma) are uniquely capable of reproducing the positive potential shift for baicalein, ffTK refinement significantly enhances the accuracy of the Charmm force field for predicting negative shifts, particularly for chrysin. Furthermore, the machine-learning-based Espaloma performed comparably to or better than GAFF2, especially for luteolin. Notably, the transition from CGenFF v.4 to v.5 did not yield substantial improvements for this set of compounds, as both versions showed similar systematic deviations from experimental trends.

**Figure 4:**
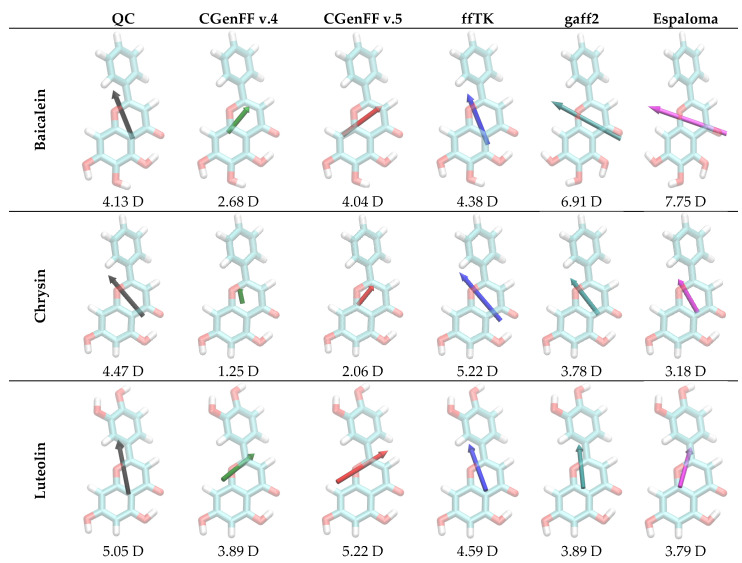
Molecular structures and dipole moment vectors of baicalein, chrysin, and luteolin across different parameterization protocols. The arrows represent the orientation and magnitude of the dipole moment calculated using quantum chemical (QC) methods (at the MP2/6-31++G** level of theory), CGenFF (v.4.6 and v.5.0), ffTK, GAFF2, and Espaloma. The corresponding dipole moment magnitudes (D, in Debyes) are indicated below each molecule.

Our previous findings demonstrated that the CGenFF v.4 protocol fails to accurately capture molecular polarity and long-range electrostatic behavior [23]. These inaccuracies are critical, as they dictate the intermolecular interactions and the preferred orientation of a modifier within the lipid bilayer. Consequently, we evaluated the dipole moment vectors across all tested parameterization strategies (Figure 4). While the systematic underestimation of dipole magnitudes in CGenFF v.4 was largely corrected in version 5.0, reaching values closer to the quantum chemical (QC) reference, the vector orientation remained inconsistent with QC for all studied molecules. This persistent directional bias explains the inability of both CGenFF versions to correctly predict changes in the membrane dipole potential.

Intriguingly, although the ffTK dipole moment for baicalein aligns closely with QC data, this protocol yielded only a marginal increase in *Ψ*, which in several replicates remained within the range of statistical fluctuation. In contrast, the Amber-based protocols successfully reproduced the experimental *Ψ* shift, despite significantly overestimating the dipole magnitude. For luteolin, both ffTK and Amber-based strategies show good agreement with experimental trends; despite their differences in dipole magnitude, their predicted vector orientations are similar. These observations imply that the accurate description of an isolated molecule’s dipole moment does not guarantee a correct prediction of its effect on membrane electrostatics. The resulting change in *Ψ* is a multi-factor process determined not only by the intrinsic molecular dipole, but also by the modifier’s specific orientation, its insertion depth, and the local restructuring of the lipid–water interface.

As presented in Table 4, a detailed analysis of the structural parameters clarifies the molecular mechanisms behind the observed dipole potential modifications. For baicalein, the structural data directly support why only the Amber-based protocols successfully reproduced the positive potential shift. Specifically, GAFF2 and Espaloma stabilize baicalein in a significantly more upright orientation, yielding the lowest tilt angles of 59.85° ± 4.79° and 50.99° ± 2.95°, respectively. Concurrently, these protocols suppress molecular aggregation, as evidenced by the minimal number of inter-flavonoid contacts (1.06 ± 0.41 for Gaff2 and 0.84 ± 0.74 for Espaloma). This combination of a perpendicular orientation and monomeric state allows the molecular dipoles to project effectively along the z-axis. In stark contrast, the ffTK refinement forces the baicalein core to lie almost completely flat on the membrane surface (85.79° ± 2.26°) while promoting severe self-association with the highest contact number in the set (2.59 ± 1.18), which completely neutralizes its electrostatic impact.

For chrysin, the predictive success of both the refined Charmm (ffTK) and Amber (GAFF2, Espaloma) protocols stems from their structural convergence. Excluding the outdated CGenFF v.4 (58.11° ± 1.95°), all these strategies consistently predict that chrysin adopts a flat orientation, with tilt angles tightly clustered between 76.18° and 79.48°. Under these protocols, the molecules also maintain a moderate and comparable degree of clustering (1.25 to 2.14 contacts). This structural consensus explains why both ffTK and GAFF2 managed to reproduce the experimental negative shift with high accuracy, as the parallel-to-surface alignment of the chrysin core dictates the correct orientation of its dipole vector within the bilayer environment.

Luteolin exhibits a distinct behavior, locating the highest within the bilayer structure with the immersion depths of 1.32 to 1.43 nm from the center. Possessing the highest number of hydroxyl groups among the studied compounds, this elevated positioning within the lipid headgroup region enables luteolin to interact more effectively with the aqueous phase. Crucially, our previous study demonstrated that the electrostatic modifications induced by luteolin are primarily driven by the restructuring of the water dipole potential, while exerting a minimal impact on the intrinsic dipole potential of the lipid membrane itself [23]. This potent interfacial effect is further amplified by two key factors: a highly parallel orientation relative to the membrane surface (tilt angles of 72.01° to 87.34°) and a remarkably low propensity for conglomeration, with inter-molecular contacts remaining below 1.0 (reaching a minimum of 0.39 ± 0.29 in Espaloma). This highly dispersed, flat alignment at a shallow depth prevents mutual dipole cancellation and optimizes the restructuring of the lipid–water interface, explaining why all tested protocols successfully identified luteolin as the most potent modifier inducing the largest negative Δ*Ψ* shift.

## 4. Conclusions

In this study, we performed a systematic evaluation of the *Ψ* sensitivity to system composition, water models, and small-molecule parameterization protocols. Our results indicate that while both force fields exhibit finite-size effects as the system scales, the Charmm (CHARMM36m) force field is notably more sensitive to minor fluctuations in box composition (hydration and ion count) compared to Amber (Lipid21). However, our statistical analysis confirms that these sensitivities do not compromise the overall reliability of the results, as a system size of 120 lipids provides sufficient convergence and high reproducibility across independent replicates. Consequently, we conclude that the 120-lipid setup is a robust and computationally efficient framework for determining the *Ψ* and investigating its modifications.

Furthermore, we demonstrated that the transition from 3-site (TIP3P) to refined 4-site water models (TIP4P-Ew and OPC) leads to a statistically significant increase in the absolute *Ψ* by approximately 100 mV. Our decomposition analysis reveals that this shift is almost entirely solvent-driven, while the lipid–water electrostatic coupling remains remarkably consistent across different water models. Importantly, since this offset is systematic and independent of the force field, the relative changes induced by dipole modifiers are preserved. These findings provide a solid rationale for employing the computationally efficient TIP3P model in high-throughput studies, as it accurately captures the *ΔΨ* shifts despite the lower absolute baseline.

The central finding of this study is the distinct performance of different parameterization protocols in predicting *Ψ* modifications. For the Charmm force field, standard CGenFF failed to reproduce experimental trends, while ffTK-refinement significantly enhanced accuracy. In the Amber framework, both GAFF2 and Espaloma successfully captured the experimental shifts. Notably, the surprisingly high accuracy of Espaloma underscores the immense potential of machine learning approaches, which offer a robust and automated alternative to traditional methods. However, from a practical workflow perspective, GAFF2 remains more advantageous as it is natively integrated into the AmberTools suite and requires no external deep learning dependencies or software environments. Based on our results, we recommend utilizing ffTK-refinement for Charmm-based studies or GAFF2 for Amber-based protocols, with a clear outlook toward ML-driven parameterization for future drug design and biophysical applications.

## Figures and Tables

**Table 1:** Mean potential of DOPC membrane system for Charmm and Amber force fields under varying simulation box composition.

Lipids	Water/Lipid	Ions	KCl	Potential (mV)	
				Charmm FF	Amber FF
120	60	20	0.1 M	629.1 ± 4.4	1267.3 ± 5.5
120	60	22	0.11 M	623.6 ± 6.6	1268.1 ± 9.2
120	65	20	0.09 M	626.1 ± 8.4	1272.1 ± 12.2
120	65	22	0.1 M	622.6 ± 4.9	1263.1 ± 18.3
60	60	10	0.1 M	620.9 ± 14.3	1274.3 ± 9.7
240	60	44	0.1 M	619.4 ± 5.8	1262.6 ± 2.5
480	60	88	0.1 M	610.4 ± 2.6	1246.8 ± 8.6

**Table 2:** Statistical summary of the Linear Mixed-Effects Model for membrane potential.

Factor	Charmm FF		Amber FF	
	Coefficient ± SE	*p*-Value	Coefficient ± SE	*p*-Value
Intercept (Base)	0.629 ± 0.003	<0.001	1.267 ± 0.006	<0.001
Hydration	−0.003 ± 0.004	0.444	0.005 ± 0.009	0.579
Ions	−0.005 ± 0.004	0.157	0.001 ± 0.009	0.925
Interaction	0.002 ± 0.005	0.725	−0.010 ± 0.012	0.419
Residual Variance	0.0233	—^#^	0.0217	—^#^
Group Variance	0.000	—^#^	0.000	—^#^
Model Convergence	Yes	—^#^	Yes	—^#^

# - there is no indicator for this value.

**Table 3:** Predicted membrane dipole potential values (mV) for systems with flavonoids using different force field parameterization protocols.

Compound	N of Molecules	Charmm Force Field			Amber Force Field	
		CGenFF v.4	CGenFF v.5	ffTK	GAFF2	Espaloma
Baicalein	12	609 ± 2	588 ± 15	637 ± 11	1288 ± 12	1319 ± 7
	24	594 ± 8	566 ± 7	628 ± 14	1316 ± 15	1377 ± 10
Chrysin	12	602 ± 8	593 ± 5	572 ± 7	1214 ± 19	1227 ± 18
	24	597 ± 8	562 ± 6	539 ± 8	1192 ± 9	1213 ± 19
Luteolin	12	519 ± 6	552 ± 11	479 ± 12	1115 ± 7	1166 ± 7
	24	436 ± 10	479 ± 4	361 ± 10	973 ± 15	1023 ± 5

**Table 4:** Structural and orientational characteristics of flavonoids in the lipid bilayer at a 10:1 lipid-to-modifier ratio.

Flavonoid	Parameterization	Immersion Depth (from Bilayer Center), nm	Tilt Angle ⟨θ⟩, deg	Number of Inter-Flavonoid Contacts (Within 6 Å)
Baicalein	CGenFF v.4	1.22 ± 0.02	66.01 ± 2.26	2.06 ± 0.51
	CGenFF v.5	1.31 ± 0.01	70.70 ± 1.56	1.75 ± 0.38
	ffTK	1.08 ± 0.02	85.79 ± 2.26	2.59 ± 1.18
	Gaff2	1.24 ± 0.01	59.85 ± 4.79	1.06 ± 0.41
	Espaloma	1.23 ± 0.01	50.99 ± 2.95	0.84 ± 0.74
Chrysin	CGenFF v.4	1.20 ± 0.01	58.11 ± 1.95	1.94 ± 0.65
	CGenFF v.5	1.33 ± 0.01	77.21 ± 2.14	2.14 ± 1.39
	ffTK	1.16 ± 0.02	76.18 ± 0.90	1.65 ± 0.43
	Gaff2	1.12 ± 0.02	78.58 ± 5.59	2.11 ± 0.79
	Espaloma	1.14 ± 0.01	79.48 ± 5.19	1.25 ± 1.14
Luteolin	CGenFF v.4	1.36 ± 0.01	87.34 ± 1.97	0.75 ± 0.49
	CGenFF v.5	1.43 ± 0.03	75.55 ± 2.21	0.67 ± 0.68
	ffTK	1.28 ± 0.01	83.21 ± 1.58	0.66 ± 0.21
	Gaff2	1.32 ± 0.01	74.12 ± 2.59	0.52 ± 0.13
	Espaloma	1.33 ± 0.03	72.01 ± 4.09	0.39 ± 0.29

## Data Availability

The original contributions presented in this study are included in the article. Further inquiries can be directed to the corresponding author.
